# Glutamate Signaling in Healthy and Diseased Bone

**DOI:** 10.3389/fendo.2012.00089

**Published:** 2012-07-19

**Authors:** Robert W. Cowan, Eric P. Seidlitz, Gurmit Singh

**Affiliations:** ^1^Department of Pathology and Molecular Medicine, McMaster UniversityHamilton, ON, Canada

**Keywords:** glutamate, bone, rheumatoid arthritis, signaling, bone disease, cancer, homeostasis, pathology

## Abstract

Bone relies on multiple extracellular signaling systems to maintain homeostasis of its normal structure and functions. The amino acid glutamate is a fundamental extracellular messenger molecule in many tissues, and is used in bone for both neural and non-neural signaling. This review focuses on the non-neural interactions, and examines the evolutionarily ancient glutamate signaling system in the context of its application to normal bone functioning and discusses recent findings on the role of glutamate signaling as they pertain to maintaining healthy bone structure. The underlying mechanisms of glutamate signaling and the many roles glutamate plays in modulating bone physiology are featured, including those involved in osteoclast and osteoblast differentiation and mature cell functions. Moreover, the relevance of glutamate signaling systems in diseases that affect bone, such as cancer and rheumatoid arthritis, is discussed, and will highlight how the glutamate system may be exploited as a viable therapeutic target. We will identify novel areas of research where knowledge of glutamate communication mechanisms may aid in our understanding of the complex nature of bone homeostasis. By uncovering the contributions of glutamate in maintaining healthy bone, the reader will discover how this complex molecular signaling system may advance our capacity to treat bone pathologies.

Glutamate (in the form of l-glutamate) is a non-essential amino acid that is the most common excitatory neurotransmitter in the central nervous system (CNS; Bleich et al., 2003). Glutamate signaling is phylogenetically ancient and common in both plants and animals (Chiu et al., 1999), likely evolving from an important role in the regulation of carbon and nitrogen metabolism (Davenport, 2002). Synthesized intracellularly in mammalian cells, glutamate is primarily formed from glutamine using the enzyme glutaminase, to produce an ammonium ion useful for excess nitrogen disposal (Young and Ajami, 2001). Transamination of α-ketoglutarate, an intermediate of the citric acid cycle, also generates glutamate (Waddell and Miller, 1991). Glutamate is widely used as an intercellular communication molecule (see reviews by Watkins and Jane, 2006; Krnjevic, 2010), and although characterized more fully in the CNS, all of the essential components for glutamate signaling have been identified in non-neural systems (Skerry and Genever, 2001; Nedergaard et al., 2002; Hinoi et al., 2004a). Moreover, a fully functional glutamate signaling system is present in bone (Spencer et al., 2007). Although numerous systemic, local, and neural factors are involved in regulating bone remodeling (Berenson et al., 2006; Elefteriou, 2008; Martin and Seeman, 2008), there is growing recognition of the importance of glutamate signaling in bone homeostasis (Nedergaard et al., 2002; Hinoi et al., 2004b; Spencer et al., 2004). Following an overview of the structural components of glutamate signaling systems, this review will evaluate how these structures are involved in maintaining bone homeostasis with a particular emphasis on their potential involvement in a variety of bone diseases. Indeed, several disease states may be potentiated by a disruption of normal glutamatergic signaling. A better understanding of glutamate intercellular communication in healthy and diseased bone will aid in determining whether these signaling components may represent viable therapeutic targets in bone disease.

## Glutamate Signaling Structures

**Figure 1:**
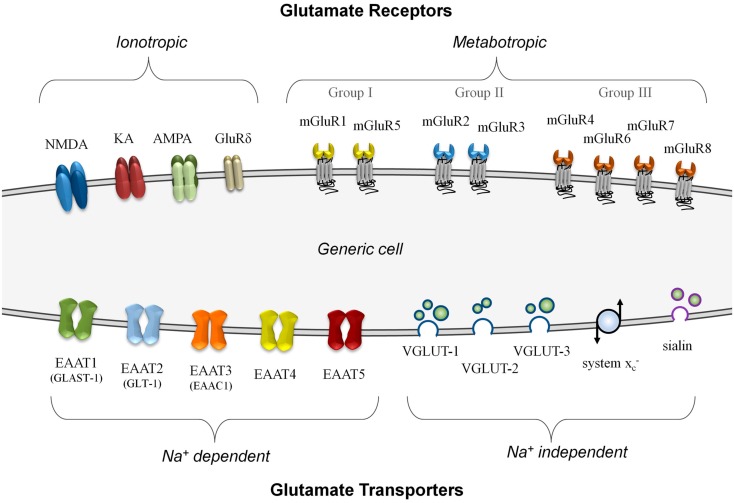
**A summary of known glutamate receptor and transporter structures**. For simplification, these structures are depicted on a generic model cell, although no cell is known to express all of these components simultaneously. Glutamate receptors are divided into either ionotropic or metabotropic types. Ionotropic receptors can incorporate different combinations of functional subunits, conferring varying response properties, and allowing for co-agonist activation. The metabotropic receptors, subdivided into three groups, are G-protein coupled membrane receptors that do not form ion channels although they can modulate other glutamate signaling components. Glutamate transporters are divided into those that are sodium-dependent and those that do not require a sodium gradient for their activity. The plasma membrane transporters (EAAT1 to EAAT5) can form ion channels and most often import glutamate. The sodium-independent transporters primarily export glutamate and these include the vesicular transporters (VGLUT-1 to VGLUT-3 and sialin) and the non-vesicular system ${\text{X}}_{\text{c}}^{–}$ glutamate/cystine antiporter.

In neurons, glutamate signaling involves several distinct steps – signal release via vesicular transporters, reception by specific receptors, and termination of the signal using uptake transporters. Each of these steps must work in concert to function effectively, and imbalances can lead to failure of the system (Olney, 1969). Figure 1 summarizes, on a generic cell, the different classes of glutamate transporters and receptors that can be involved in glutamate signaling.

There are two main categories of glutamate transporters – the predominantly vesicular glutamate *export* transporters which are sodium-independent, and the non-vesicular plasma membrane glutamate *import* transporters which are sodium-dependent. There are four types of vesicular export transporters – namely VGLUT-1 to VGLUT-3 (Hayashi et al., 2003; Tremolizzo et al., 2006; Liguz-Lecznar and Skangiel-Kramska, 2007) and sialin, a recently characterized vesicular glutamate/aspartate transporter (Miyaji et al., 2010). Although non-vesicular, the system ${\text{X}}_{\text{c}}^{–}$ glutamate/cystine antiporter is a membrane-bound glutamate exporter, and exchanges intracellular glutamate for extracellular cystine (Bannai, 1986). This amino acid exchange accumulates cystine to synthesize the antioxidant glutathione (Kim et al., 2001). For glutamate import, the excitatory amino acid transporters (EAATs) are critically important for the termination of glutamate – a good review of their properties has been prepared by Tzingounis and Wadiche (2007). There are five known non-vesicular reuptake transporters, named EAAT1 to EAAT5 (Shigeri et al., 2004). The neuronal EAAT1 transporter is also called the glutamate-aspartate transporter (GLAST-1; Huggett et al., 2002) due to its ability to transport both amino acids, while the human EAAT2 transporter known as glutamate transporter-1 (GLT-1) is often expressed in glial cells and pre-synaptic neurons (Mason et al., 1997). Although both GLAST-1 and GLT-1 primarily function as importers of glutamate, they are also capable of glutamate export under strong potassium gradients (Marcaggi et al., 2005).

One of the advantages of using glutamate for signaling is the incredible diversity of responses that can result from this molecule. Response flexibility is accomplished by an array of glutamate receptor types that can generate different responses depending on how they are expressed. Glutamate receptors are divided into two major groups on the basis of their mode of action – ionotropic glutamate receptors (iGluRs), which form ion-gated cation channels when activated, and metabotropic glutamate receptors (mGluRs), which are coupled to intracellular G-proteins and regulate the production of intracellular second messengers. An excellent review by Dingledine et al. (1999) provides a comprehensive overview of glutamate receptors and their functions, primarily within the CNS. Briefly, there are three main groups of iGluRs named according to their synthetic agonist specificity: *n*-methyl-d-aspartate (NMDA) receptors, α-amino-3-hydroxy-5-methyl-4-isoxazolepropionic acid (AMPA) receptors, and kainate (KA) receptors. Although not widely known, a fourth family of iGluRs termed the delta receptors exists, with two subtypes identified (GluRδ1 and GluRδ2), although they may only be secondarily involved in glutamate signaling (Kakegawa et al., 2011; Tanahashi et al., 2012). The iGluRs are comprised of tetramers of different subunits in various combinations that form ion channels with distinct properties. There are eight different mGluRs (mGluR1 to mGluR8) that are divided into three groups based on sequence homology and their signal transduction pathways (Niswender and Conn, 2010). Providing both excitatory and inhibitory responses, the mGluRs often modulate other systems via their second messenger activation and complex interactions with other proteins (Enz, 2007).

## Glutamate Signaling in Healthy Bone

Considerable evidence exists to suggest that glutamate signaling in normal bone is involved in a variety of processes, including cell differentiation effects and mature cell functions. Glutamate concentrations within the bone environment are modulated by a variety of cell types, through glutamate-specific transporters which are expressed by the bone-resorbing osteoclasts (Oc; Hinoi et al., 2007), bone-building osteoblasts (Ob; Takarada-Iemata et al., 2011; Uno et al., 2011), osteocytes (Huggett et al., 2002), and chondrocytes (Wang et al., 2006). In fact, virtually all cell types in normal bone have the ability to secrete glutamate to some extent through multiple mechanisms. The amount of glutamate available for release may be controlled through enzymatic processes. For example, glucocorticoids and Wnt signaling may inversely regulate glutamine synthetase activity in osteoblasts (Olkku et al., 2004; Olkku and Mahonen, 2008), which catalyzes the conversion of glutamate to glutamine.

A variety of glutamate receptors and subunit combinations are expressed within the bone environment, enabling a very high degree of control over the cellular responses to glutamate signals. The most well-characterized glutamate receptors in normal bone are the NMDA-type receptors, with confirmed expression in Oc, Ob, osteocytes, and bone marrow mononuclear cells (Chenu et al., 1998; Patton et al., 1998; Genever et al., 1999; Itzstein et al., 2001). Inhibition of these receptors prevents early differentiation of both Ob (Hinoi et al., 2003; Lin et al., 2008) and Oc (Peet et al., 1999; Merle et al., 2003; Lin et al., 2008) into their functionally active cell types. However, an inhibitor of system ${\text{X}}_{\text{c}}^{–}$, which reduces glutamate release without acting on the receptors, was also able to inhibit Oc differentiation (Suematsu et al., 2007). This implies that glutamate released by other cells within the bone may signal for the generation of new Oc or Ob via NMDA receptors. Moreover, glutamate has been shown to suppress Ob cell proliferation at the very earliest stages of differentiation from their mesenchymal stem cell progenitors, which express multiple types of glutamate receptors and transporters themselves (Iemata et al., 2007). Other receptor types are also expressed by normal bone cells, including metabotropic (Gu and Publicover, 2000; Hinoi et al., 2001; Szczesniak et al., 2005; Kalariti et al., 2007) and non-NMDA ionotropic receptors such as KA and AMPA receptors (Chenu et al., 1998; Hinoi et al., 2002; Taylor, 2002; Szczesniak et al., 2005). Table 1 summarizes the glutamate receptors and transporters expressed by normal bone cells. Deletion of osteoblastic NMDA receptor expression as well as targeted administration of iGluR antagonists were reported to impede skeletal development in mouse models (Skerry, 2008). Indeed, the AMPA receptors may also participate in Ob differentiation, as stimulation of this receptor increases mineral deposition and osteocalcin expression (Lin et al., 2008).

**Table 1:** **A summary of glutamate signaling components expressed by normal bone cells**.

			Osteoclast	Osteoblast	Osteocyte	Marrow cells	Chondrocyte
**GLUTAMATE RECEPTORS**							
Ionotropic		NMDA	**+**	**+**	**+**	**+**	**+**
		KA	**+**	**+**			
		AMPA	**+**	**+**	**±**	**±**	**+**
		GluRδ					
Metabotropic	I	mGluR1		**+**			
		mGluR5	**+**	**+**		**+**	
	II	mGluR2		**±**		**+**	
		mGluR3	**+**	**+**			
	III	mGluR4	**+**	**±**		**+**	
		mGluR6	**+**	**±**			
		mGluR7	**+**	**±**		**+**	
		mGluR8	**+**	**+**			
**GLUTAMATE TRANSPORTERS**							
Na^+^ dependent		EAAT1 (GLAST-1)	**−**	**+**	**+**		**+**
		EAAT2 (GLT-1)	**+**	**+**		**+**	**+**
		EAAT3 (EAAC1)	**−**	**+**			**+**
		EAAT4	**+**	**±**			
		EAAT5	**−**	**−**			
Na^+^ independent		VGLUT-1	**+**	**+**			
		VGLUT-2	**−**	**−**			
		VGLUT-3	**−**	**−**			
		System ${\text{X}}_{\text{c}}^{–}$	**+**	**+**			**+**
		Sialin					

*This table summarizes published reports of protein and/or RNA expression of glutamate receptors and transporters and demonstrates how they are differentially expressed in primary or cultured bone cells. Most cell types in the bone (potentially all) express the structures to release, receive, and terminate glutamate signals through multiple mechanisms (+, expressed; **−**, not expressed; ±, inconclusive; blank, unknown/not evaluated)*.

Glutamatergic signaling components are also involved in the adult functions of bone cells. In particular, Oc transport bone degradation products from the bone surface in vesicular structures and remove them from the apical end of the cell in a process called transcytosis (Yamaki et al., 2005). This essential transportation function interacts directly with VGLUT-1, which adds glutamate to the transcytotic vesicles containing the bone degradation products and upon release by the Oc, glutamate acts in an autocrine manner to suppress further bone resorption, likely through mGluR8 (Morimoto et al., 2006). However, regulation of bone resorption is also impaired by inhibitors of ionotropic receptors (Chenu et al., 1998; Peet et al., 1999; Itzstein et al., 2000), further demonstrating the complexity of this regulatory system.

To prevent continued signaling between bone cells, extracellular glutamate concentrations are reduced by plasma membrane transporters. The most studied transporter in this regard is GLAST-1, and it is constitutively expressed by Ob and osteocytes (Mason et al., 1997; Huggett et al., 2002; Mason and Huggett, 2002; Kalariti et al., 2007; Spencer et al., 2007), but not by Oc. However, Ob and osteocytes are not the only cells capable of sequestering free glutamate, as GLT-1 is also expressed by Oc, chondrocytes, and mononuclear bone marrow cells (Mason et al., 1997; Hinoi et al., 2005b, 2007; Spencer et al., 2007). Moreover, EAAT3 and EAAT4 are also expressed by Ob (Takarada et al., 2004) and Oc (Hinoi et al., 2007), respectively.

## Glutamate Signaling in Diseased Bone

Owing to their participation in normal bone cell functioning, disruption of the glutamatergic signaling mechanisms may lead to various disease pathologies. For example, use of a specific group II/III mGluR antagonist stimulates *in vitro* bone resorption by Oc compared to untreated controls (Morimoto et al., 2006). This effect is thought to result from inhibition of mGluR8, which in turn may prevent glutamate from participating in the negative feedback cascade produced by transcytosis, thereby resulting in continued Oc resorption. As mentioned, intracellular glutamate is added to the transcytotic vesicles by VGLUT-1 as part of this Oc activity feedback mechanism, and correspondingly, VGLUT-1 knockout mice develop osteoporosis due to increased bone resorption (Morimoto et al., 2006). Moreover, differential expression of NMDA receptor subunits in chondrocytes may promote osteoarthritis (Ramage et al., 2008), and glutamate signaling via NMDA receptors on osteoarthritic chondrocytes can mediate inflammatory responses (Piepoli et al., 2009).

Notably, bone homeostasis may be indirectly disrupted by glutamatergic signaling in non-bone cells as well. For example, NMDA receptors present within the parathyroid glands or kidneys may contribute to altered secretion of parathyroid hormone (Parisi et al., 2009, 2010), which can lead to both bone resorption and bone formation. Moreover, glutamate potently stimulates secretion of the hormone leptin from white adipocytes (Cammisotto et al., 2005), which, in turn, can inhibit bone formation by signaling through the sympathetic nervous system (Ducy et al., 2000; Takeda et al., 2002). Additionally, glutamate itself may affect neuronal control of bone mass, as monosodium glutamate-sensitive neurons can stimulate bone formation (Elefteriou et al., 2003).

### Cancer

Cancers are perhaps the most significant disease to result in disruption of glutamatergic signaling. Indeed, glutamate is an established contributor to the pathophysiology of gliomas (see de Groot and Sontheimer, 2011 for a recent review). However, glutamatergic signaling components are also present in multiple cancers (Stepulak et al., 2009; Sharma et al., 2010), signifying the fundamental importance of glutamate in many biologic systems. Glutamate itself may also directly participate in cancer initiation, as melanocytes from transgenic mice induced to express mGluR5 subsequently developed into melanomas (Choi et al., 2011).

With respect to bone, numerous cancers that metastasize to bone were demonstrated to express several glutamate receptors and transporters, and these included breast, prostate, and lung cancers (Rzeski et al., 2001; Narang et al., 2003; Doxsee et al., 2007; Sharma et al., 2010). Limited information is available on the participation of glutamate signaling in primary bone cancers, although human osteosarcoma cell lines express several glutamate receptors (Genever and Skerry, 2001; Itzstein et al., 2001; Kalariti et al., 2004, 2007) and GLAST-1 (Kalariti et al., 2004, 2007). NMDA receptor subunits were also identified in Oc-like giant cells from giant cell tumor of bone (Itzstein et al., 2001). Glutamate is likely important for proper tumor cell functioning, as inhibition of glutamate receptors can limit cell growth in many cancer cell lines (Rzeski et al., 2001). Conversely, pharmacologic prevention of glutamate release in osteosarcoma cells results in an inhibition of differentiation and increased apoptosis (Genever and Skerry, 2001). Therefore glutamate signaling in general may represent an important target for bone metastasis and primary tumor treatments, particularly since bone is uniquely sensitive to altered extracellular glutamate (Seidlitz et al., 2010a).

As an excitatory amino acid, glutamate may also contribute to nociception resulting from bone cancers. Indeed, in a mouse model, sarcoma cells injected into the medullary cavity of the distal femur stimulated behavioral changes indicative of bone cancer pain and increased the expression of mGluR3 and mGluR5 in the spinal cord (Ren et al., 2012). Stimulation of mGluR3 or inhibition of mGluR5 in the CNS reduced bone cancer pain, suggesting differential expression of these receptors in the spinal cord may amplify nociceptive signaling (Ren et al., 2012). Moreover, a similar model demonstrated increased expression of the NMDA receptor subunit NR2B in the spinal cord, and inhibition of NMDA receptor activity decreased pain symptoms (Gu et al., 2010). However, glutamate from non-neural sources may also stimulate nociceptors in bone, and cancer cells release significant amounts of glutamate via the system ${\text{X}}_{\text{c}}^{–}$ glutamate/cystine antiporter (Seidlitz et al., 2009; Sharma et al., 2010). This transporter is an especially attractive therapeutic target in cancer as inhibiting its functions can increase sensitivity of the cancer cell to oxidative stress. An excellent review by Lo et al. (2008) discusses system ${\text{X}}_{\text{c}}^{–}$ inhibition and its potential to limit tumor growth and sensitize cancers to other treatments largely through pharmacologic inhibition of cystine intake, which leads to inadequate glutathione production and an increased sensitivity to oxidative stress. Excess glutamate may further disrupt the bone by interfering with the normally balanced Oc and Ob intercellular signaling, as a moderate increase in extracellular glutamate was able to increase mineralized bone formation in cultured Ob (Seidlitz et al., 2010b). In this respect, therapeutic inhibition of system ${\text{X}}_{\text{c}}^{–}$ in the cancer cells may relieve bone pain symptoms by reducing their glutamate secretion while simultaneously preventing tumor growth, reducing bone disruption, and sensitizing the tumor to radiation or chemotherapeutic agents.

### Rheumatoid arthritis

Glutamate signaling is also relevant in rheumatoid arthritis (RA). For example, glutamate concentrations in synovial fluid were reported to increase more than 50-fold in patients with RA compared to controls, from 6.25 to 326 μM (McNearney et al., 2000) and both glutamate receptors and transporters are expressed by synovial fibroblasts (Hinoi et al., 2005a; Flood et al., 2007). RA, a chronic disorder characterized by synovial inflammation, can result in degradation of cartilage and bone by the invading pannus. As with cancer, excess glutamate within the synovial fluid may correspond with increased nociception (Sluka et al., 1994; Lawand et al., 1997). Glutamate signaling is also thought to contribute to the inflammatory response, as a KA receptor antagonist decreases expression of interleukin-6 in primary synovial fibroblasts (Flood et al., 2007), and exogenous glutamate was found to stimulate tumor necrosis factor-α expression in primary synovial cell cultures established from RA patients (McNearney et al., 2004). NMDA receptor antagonists also increase expression of pro-matrix metalloproteinase 2 in RA synovial fibroblasts (Flood et al., 2007). Moreover, blood glutamate concentration is associated with increased bone resorption in RA patients (Hajati et al., 2009, 2010). Not surprisingly, therefore, modulation of glutamate signaling may alleviate RA symptoms. It is intriguing to note that a common treatment for RA is sulfasalazine, and this drug is particularly effective at inhibiting the system ${\text{X}}_{\text{c}}^{–}$ glutamate/cystine antiporter (Doxsee et al., 2007). Indeed, the importance of glutamate in arthritis is further highlighted using rodent arthritis models, as inhibition of NMDA with memantine delayed the onset of collagen-induced arthritis and reduced bone resorption in mice (Lindblad et al., 2012). The use of non-NMDA ionotropic receptor antagonists (Sluka et al., 1994), or a combination of NMDA and non-NMDA ionotropic receptor antagonists (Lam and Ng, 2010) also reduced swelling and alleviated pain symptoms in rat early arthritis models.

## Conclusion

Healthy bone is maintained in an exquisitely balanced state by numerous intercellular communication systems. Evidence that glutamate signaling is a significant participant in bone homeostasis continues to accumulate, and disruptions in glutamatergic mechanisms may contribute to a variety of bone diseases. However, owing to their apparent fundamental importance in numerous organ systems, future *in vivo* studies will require targeted manipulation of glutamatergic signaling to evaluate such consequences on bone homeostasis. Bone may be especially susceptible to glutamate interference as bone cells express the necessary receptors and transporters to transmit and receive glutamate signals for their normal functions. Glutamate transport appears critical for feedback control between Oc and Ob and disruption of this process may be relevant in osteoporosis. In osteoarthritis, glutamate receptor expression is altered compared to normal bone cells, and extracellular glutamate concentrations are significantly increased in affected joints in RA and could impact inflammatory responses. In arthritis, glutamate-sensitive nociceptors may be stimulated by this locally elevated signal, and may contribute to arthritis pain. Cancers growing in bone significantly disrupts bone metabolism and causes severe pain. As cancer cells are known to secrete glutamate, this errant extracellular signal may deregulate the tightly coupled glutamatergic bone remodeling process and may directly stimulate nociceptors. Pharmacologic modulation of the transporter secreting glutamate from cancer cells is offered as a potential strategy to reduce both metabolic disruption and pain in bone cancer, and in a wider context, suggests that the glutamatergic communication system holds significant potential as a therapeutic target in a number of bone-related disorders. As glutamate signaling is such a fundamental biological process, identifying how it functions in normal bone is vital to advancing our capacity to develop glutamate-based treatments for bone diseases.

## Conflict of Interest Statement

The authors declare that the research was conducted in the absence of any commercial or financial relationships that could be construed as a potential conflict of interest.
